# SMG‐1 inhibition by miR‐192/‐215 causes epithelial‐mesenchymal transition in gastric carcinogenesis via activation of Wnt signaling

**DOI:** 10.1002/cam4.1237

**Published:** 2017-12-13

**Authors:** Xiaojing Zhang, Yin Peng, Yong Huang, Mengting Yang, Ruibin Yan, Yanqiu Zhao, Yulan Cheng, Xi Liu, Shiqi Deng, Xianling Feng, Huijuan Lin, Huimin Yu, Si Chen, Zhenfu Zhao, Shanni Li, Kuan Li, Liang Wang, Yanjie Wei, Zhendan He, Xinmin Fan, Stephen J. Meltzer, Song Li, Zhe Jin

**Affiliations:** ^1^ Department of Pathology The Shenzhen University School of Medicine Shenzhen Guangdong 518060 China; ^2^ Shenzhen Key Laboratory of Micromolecule Innovatal Drugs Shenzhen Key Laboratory of translational Medicine of Tumor, Guangdong Key Laboratory for Genome Stability & Disease Prevention Shenzhen University School of Medicine Shenzhen, Guangdong China; ^3^ Department of Pathology Wuhan University School of Basic Medical Sciences Hubei China; ^4^ Laboratory of Chemical Genomics The Shenzhen Graduate School of Peking University Shenzhen Guangdong China; ^5^ Department of Medicine/GI Division Johns Hopkins University Sidney Kimmel Comprehensive Cancer Center Baltimore Maryland; ^6^ Department of Pathology and Pathophysiology The Guangzhou Medical University Guangzhou Guangdong China; ^7^ Center for High Performance Computing Shenzhen Institutes of Advanced Technology Shenzhen Guangdong China; ^8^ Guangdong Province Key Laboratory of Molecular Oncologic Pathology Guangzhou Guangdong China

**Keywords:** EMT, gastric cancer, miR‐192/‐215, SMG‐1, Wnt signaling pathway

## Abstract

SMG‐1,a member of the phosphoinositide kinase‐like kinase family, functioned as a tumor suppressor gene. However, the role of SMG‐1 in GC remain uncharacterized. In this study, regulation of SMG‐1 by miR‐192 and‐215, along with the biological effects of this modulation, were studied in GC. We used gene microarrays to screening and luciferase reporter assays were to verify the potential targets of miR‐192 and‐215. Tissue microarrays analyses were applied to measure the levels of SMG‐1 in GC tissues. Western blot assays were used to assess the signaling pathway of SMG‐1 regulated by miR‐192 and‐215 in GC. SMG‐1 was significantly downregulated in GC tissues.The proliferative and invasive properties of GC cells were decreased by inhibition of miR‐192 and‐215, whereas an SMG‐1siRNA rescued the inhibitory effects. Finally, SMG‐1 inhibition by miR‐192 and‐215 primed Wnt signaling and induced EMT. Wnt signaling pathway proteins were decreased markedly by inhibitors of miR‐192 and‐215, while SMG‐1 siRNA reversed the inhibition apparently. Meanwhile, miR‐192 and‐215 inhitibtors increased E‐cadherin expression and decreased N‐cadherin and cotransfection of SMG‐1 siRNA reversed these effects. In summary, these findings illustrate that SMG‐1 is suppressed by miR‐192 and‐215 and functions as a tumor suppressor in GC by inactivating Wnt signaling and suppressing EMT.

## Introduction

SMG‐1(Suppressor with morphogenetic effect on genitalia family member 1), the most recently discovered member of the phosphoinositide kinase‐like kinase (PIKK) family, is involved in the degradation of nonsense‐mediated mRNA decay (NMD) in *Caenorhabditis elegans* and mammalian cells. Additionally, SMG‐1 plays other cellular roles, such as regulation of the G1/S checkpoint, response to hypoxia, response to IR and UV radiation, cell growth, and stress responses 1. Recently, SMG‐1 was shown to exhibit tumor‐suppressive properties. For example, Gubanova et al. 2 showed that SMG‐1 suppressed oncogenic CDK2‐driven proliferation and was a tumor suppressor in osteosarcoma. Similarly, in human papillomavirus (HPV)‐positive head and neck squamous cell carcinoma, SMG‐1 was underexpressed and exhibited tumor‐suppressive activity 3. However, thus far, the precise mechanisms of participation of SMG‐1 in human carcinogenesis remain unclear.

Gastric cancer (GC) remains one of the most lethal malignancies worldwide. GC accounts for nearly 42% of all cancer cases in China 4. Despite advances in surgical, chemotherapeutic, and radiotherapeutic advances, 5‐year survival rates have improved very little. Although oncogenes and tumor suppressor genes have been identified in GC, this disease is still a major clinical problem in China. Moreover, molecular mechanisms underlying GC are poorly understood. Therefore, potential mechanistic pathways and biomarkers of GC should be researched urgently.

MicroRNAs (miRs) bind to their target mRNA 3′‐UTR sequences through a “seed sequence”, leading to target mRNA degradation or inhibition of protein translation 5. MiR‐192 and ‐215 were formerly studied by us, and both have been reported to be dysregulated in multiple cancers, including GC, renal childhood neoplasms, and colorectal cancer 6, 7, 8. In our previous study, we also showed that miR‐192 and ‐215 were upregulated and functioned as oncogenic miRs in GC 5. In a subsequent study, SMG‐1 was shown to be a target of miR‐192 and ‐215. Therefore, we further characterized the involvement of SMG‐1 in gastric carcinogenesis, including its inhibition by miR‐192 and ‐215.

In this study, we investigated the effect of SMG‐1 on GC cell proliferation, migration and invasion. We investigated whether Wnt was involved in biological activities of SMG‐1 in the context of GC. Finally, we assessed whether SMG‐1 expression correlated with clinical parameters in GC patients. Our data now suggest that SMG‐1 may represent a therapeutic target in GC.

## Materials and Methods

### Cell lines, human tissue samples, and animals

HFE145 was obtained from Howard University (Dr Duane T Smoot). Human GC cell lines BGC‐823 was obtained from Cell Bank of the Chinese Academy of Sciences (Shanghai, China). The cells were cultured in DMED (Hyclone, USA), supplemented with 10% (v/v) fetal bovine serum (FBS).The cells were kept in an incubator under 5% CO_2_ at 37°C.

Fresh GC samples were obtained from patients without prior radiotherapy and chemotherapy at the Department of general surgery of the first Affiliated Hospital of Shenzhen University, Shenzhen, China. Tissues were saved immediately in RNAlater (Ambion, USA) after resection, and then stored at −80°C until needed. For the use of these clinical materials for research purposes, prior patient's consent and approval from the Institute Research Ethics Committee were obtained.

Four‐to‐six‐week‐old female athymic BALB/c‐nu/nu mice were purchased from the Laboratory Animal Central of Guangdong Province (Guangdong, China), and maintained in a SPF(specific Pathogen Free) environment. All protocols for animal studies were reviewed and approved by the Institutional Animal Care and Use Committee of Medical College of Shenzhen University.

### Gene microarrays

To screening the potential targets of miR‐192 and ‐215, gene microarrays were carried out on the Agilent Whole Genome Oligo Microarrays (4x44K, Agilent, Santa Clara, CA, USA) in GC cells. All the procedures were referred as the manufacture protocols. Briefly, microarrays performance and analysis were performed on two groups of cell lines: BGC823 cells with loss‐function of miR‐192 and ‐215, and HFE145 cells with gain‐function of miR‐192 and ‐215. Lyse cells directly in a culture dish by adding 1 mL of TRLzol Reagent (Invitrogen, Carlsbad, California, USA) to a 3.5 cm diameter dish, and passing the cell lysate several times through a pipette. The amount of TRIzol Reagent added is based on the area of the culture dish (1 mL per 10 cm^2^). The quality and quantity of the RNA samples were checked, using an ND‐1000 Spectrophotometer (NanoDrop Technologies, Wilmington, Delaware, USA). RNA Integrity and cDNA contamination were tested by Denaturing Agarose Gel Electrophoresis. Agilent Quick Amp Labeling Kit and Agilent's Sure Hyb Hybridization Chambers (Agilent, Santa Clara, CA, USA) were used for sample labeling and hybridization, respectively. After scanning, data were extracted and further analysis was performed, using Agilent GeneSpring GX 11.5.1 software.

Upregulated and downregulated genes were identified by the threshold with at least twofold between the two groups as significance.

### Reagents, transfection and construction of plasmids

Synthesized miR‐192 and ‐215 mimics, inhibitors and corresponding nonspecific sequences (negative control, NC) for in vitro study were purchased from Dharmacon (Lafayette, CO, USA). Cholesterol‐conjugated miR 5‐192 and ‐215 inhibitors and SMG‐1 siRNA for in vivo RNA delivery, and their respective negative controls were from Ribobio Co. (Guangzhou, China).

Here, 50% confluent cells were transfected with 60 nM of each miR, using Lipofectamine RNAi MAX (Invitrogen). For depletion of SMG‐1, human SMG‐1 siRNA (siRNA1, siRNA2, and siRNA3 sequences were listed in the Table) (Guangzhou, Ribobio, Co., Ltd) were transfected into BGC823 cells. A scramble siRNA, which has no homology with the mammalian mRNA sequence was used as control.

For Luciferase activity assay, SMG‐1‐3′UTR segments and SMG‐1‐3′UTR‐mutant segments containing putative miR‐192 and ‐215 binding site were inserted into psiCHECKTM‐2 vector (Promega, USA). Primer sequences were as the Table S1. For the co‐transfection of miR and Luciferase report vectors, we transfected 60 nmol/L of miR and 40 ng of plasmids, using Lipofectamine 3000 according to the instructions (Invitrogen, USA).

### Luciferase activity assay

For 3′UTR luciferase reporter assays, we co‐transfected the luciferase vectors and miR‐192 and ‐215, using Lipofectamine 3000 (Invitrogen). The psiCHECKTM‐2‐SMG‐1‐3′UTR(wide type, WT) and psiCHECKTM‐2‐SMG‐1‐3′UTR‐mutant (mutation type, Mut) were co‐transfected with miR‐192 and ‐215 into cells. Inhibitors and mimics of miR‐192 and ‐215 were co‐transfected with Luc reporters into BGC823 and HFE145 cells, respectively. Luciferase activities were measured 48 h after transfection by the Dual‐Luciferase Reporter Assay Kit (Promega). Luminescence intensity was measured by Infinite M200 pro fluorometry (TECAN, Switzerland), and the luminescence intensity of firefly luciferase was normalized to that of Renilla luciferase. Each assay was repeated in three independent experiments.

### Tissue microarrays and immunohistochemistry

Tissue microarrays (HStm‐Ade180Sur‐06) were purchased from Outdo Biotech (Shanghai Outdo Biotech Co., LTD), which contained paired GC and para‐cancer tissues from 90 GC patients. All patients were followed up for 5–6 years and their complete clinicopathology data were collected for further analysis. The EnVision+ detection system (Dako) was used following the manufacturer's instructions. The stained tissue sections were reviewed and scored separately by two pathologists blinded to the clinical parameters. Immunostained microarrays were scored by multiplying the intensity (0–3) of staining.

### Western blotting

Cells were washed twice with cold phosphate buffered saline (PBS) and lysed in Laemmli sample buffer (Bio‐Rad) with a protease inhibitor, completely EDTA free (Roche). Protein concentration was estimated, using a BCA Protein Assay kit (Pierce, Rockford, MA, USA). Here, 30 μg proteins were electrophoresed on 6% denaturing SDS polyacrylamide gel, and transferred to polyvinylidene fluoride membranes (ImmobilonP; Millipore, Bedford, MA, USA). The membranes were blocked with nonfat milk and 0.1% Tween 20 in Tris‐buffered saline, then, incubated with the primary antibodies overnight at 4°C. The primary antibodies were as the following: SMG‐1(SIGMA, USA), *β*‐actin(Cell Signaling Technology, Inc, USA), Wnt/*β*‐Catenin Activated Targets Antibody Sampler Kit(Cell Signaling Technology, Inc, USA) and Epithelial‐Mesenchymal Transition (EMT) Antibody Sampler Kit(Cell Signaling Technology, Inc, USA). Secondary antibodies were incubated at room temperature for 1 hour. Signals were detected by enhanced chemiluminescence (Pierce, Rockford, IL, USA).

### Proliferation, plate colony formation, scratch assay, cell cycle, and cell invasion assays in vitro

The proliferation, cell cycle, and invasion assays were performed according to established protocols 5. Plate colony formation aims to detect the growth of cells, 200 cells were planted in each well of a 6‐well plate. Thereafter, 2 weeks later, the cell colonies were stained using Giemsa solution. The colony containing more than 50 cells was counted and the number of colonies was calculated. The colony formation rate was calculated by the equation: Colony formation rate = (Number of colonies/Number of seeded cells) × 100%. For the scratch assay, the same number of BGC823 cells were cultured in a 6‐well plate. When cells grew to the level of 90% confluence, 200 *μ*L pipette tips were used to scratch, and PBS was used to remove floating cells. Cells were further incubated in DMEM supplemented with 10% FBS. At 24, and 48 h, we would have taken the images with an Olympus light microscope.

### Animal experiment

All animal experiments were undertaken in accordance with the National Institute of Health Guide for the Care and Use of Laboratory Animals, with the approval of the Scientific Investigation Board of Shenzhen University, Shenzhen. To evaluate in vivo tumor growth, 1 × 10^6^ BGC823 cells were injected subcutaneously into the flank of nude mice, left or right flank, respectively (*n* = 5 per group). For delivery of cholesterol‐conjugated RNA, 10 nmol RNA in 0.1 mL saline buffer was locally injected into the tumor mass once every 3 days for 2 weeks. There were three groups as in the following: inhibitor negative control (NC), miR‐192 inhibitor (192‐inh), and miR‐215 inhibitor (215‐inh). Tumors were measured to estimate volume from day 5 to day 28 after injection. The tumor volume (mm^3^) was calculated according to the formula: volume (mm^3^ = 1/2 × length × width^2^).

### Statistical analysis

All statistical analyses were performed by SPSS 13.0 statistical software, which was considered to be significant when *P* < 0.05. Statistical significances among/between groups were tested, using one‐way ANOVA. The correlation between the expression of SMG‐1 and various clinicopathological parameters was evaluated with chi‐square test. Survival analyses were performed according to the Kaplan–Meier method and compared by the log‐rank test. Cell proliferation, cell cycle and invasion assays in vitro were all tested by one‐way ANOVA.

## Results

### SMG‐1 is a target of miR‐192 and ‐215 in GC

**Figure 1:**
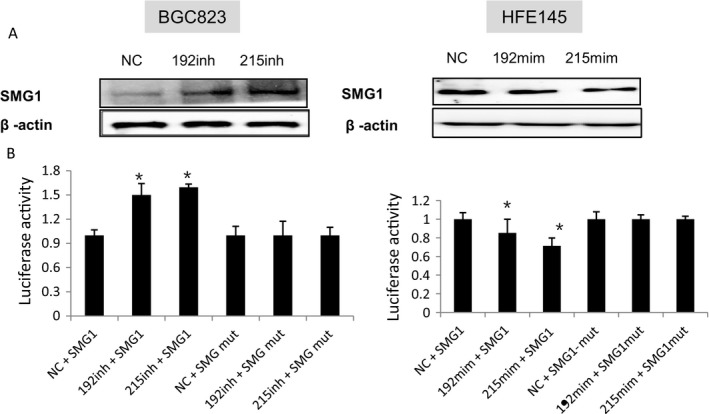
SMG‐1 is the target of miR‐192 and ‐215. Cells were transfected with miR‐192 and ‐215 inhibitors or with miR‐192 and ‐215 mimics. (A) Western blotting showed that mimics of miR‐192 and ‐215 inhibited expression of SMG‐1 in HFE145 cells, whereas inhibition of expression of miR‐192 and ‐215 stimulated SMG‐1 levels in BGC823 cells. (B) Luciferase activities of WT 3′UTR SMG‐1‐luc and MUT 3′UTR SMG‐1‐luc constructs in BGC823 and HFE145 cells. Luciferase reporter activities were significantly decreased or increased by mimics or inhibitors of miR‐192 and ‐215, respectively. Data represent mean ± SD. *P < 0.05. Assays were repeated three times, with triplicates performed in each independent experiment. SMG1,SMG‐1; 192,miR‐192;215,miR‐215;NC, nonsense control; mim, mimic; inh, inhibitor.

To discover targets of miR‐192 and ‐215 that are significantly dysregulated in GC, cDNA microarray assays was performed. HFE145 and BGC823 cells were transfected with mimics or inhibitors of miRs‐192 and ‐215, or a scrambled negative control (NC), respectively. Based on these analyses, SMG‐1 was selected due to showing greater than a twofold change in expression. Results showed that SMG‐1 was downregulated in threefold change transfected with miR‐192 mimic and fourfold change with miR‐215 mimic, respectively. On the contrary, inhibition of miR‐192 and ‐215, SMG‐1 was upregulated in sixfold and fourfold change, respectively (Table S2). Simultaneously, *in silico* searches, using miRanda (http://www.microrna.org/ microrna/home.do) and PicTar (http://pictar.bio.nyu.edu/), identified SMG‐1 as a predicted target of miR‐192 and ‐215. Finally, Western blotting assays confirmed that protein levels of SMG‐1 were induced by inhibitors and suppressed by mimics of both miR‐192 and ‐215 in gastric cells. MiR‐192 and ‐215 mimics suppressed SMG‐1 expression in HFE145 cells, while miR‐192 and ‐215 inhibitors stimulated SMG‐1 expression in BGC823 cells (Fig. 1). To establish a direct targeting relationship between miRs‐192/‐215 and SMG‐1, luciferase reporter assays were performed. Transient transfection of WT SMG‐1‐luc reporter with miR‐192 and ‐215 mimics into HFE145 cells significantly decreased luciferase activity compared with MUT control (*P *<* *0.05). In contrast, luciferase activity of the SMG‐1 reporter was enhanced in BGC823 cells transfected with miR‐192 and ‐215 inhibitors (*P *<* *0.01, Fig. 1). These results show that SMG‐1 is inhibited by miR‐192 and ‐215 and is a direct downstream target of these miRs.

**Figure 2:**
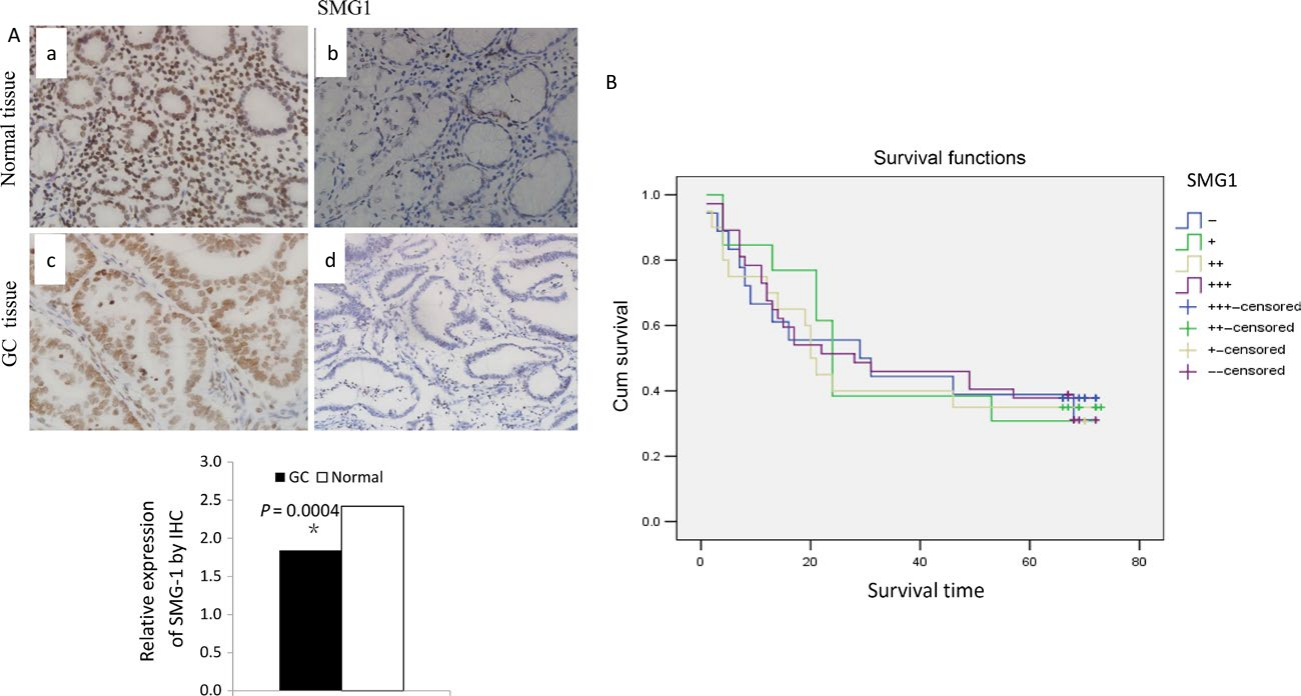
SMG‐1 protein expression in normal mucosa and GC. (a) Expression of SMG‐1 in gastric cancer (GC) tissues and surrounding noncancerous tissues by immunohistochemistry (IHC). (A) Strongly positive expression of SMG‐1 in normal gastric tissues. (b) Weak expression of SMG‐1 in normal gastric tissues. (c) Strong expression of SMG‐1 in GC tissues. (d) Negative expression of SMG‐1 in GC tissues. The bar graph shows results of paired t‐testing comparing SMG‐1 protein levels in GC tissues *vs*. matching noncancer tissues. Data represent the means. (B) Kaplan–Meier survival analysis of SMG‐1 in GC patients. This analysis shows that SMG‐1 protein level was not a significant prognostic factor in GC patients.

Next, expression of SMG‐1 was measured by IHC on a GC tissue microarray. The tissue microarray contained 90 paired GC and para‐cancer normal tissues, with accompanying clinical annotation, representing 5 years of follow‐up data. SMG‐1 staining was principally observed in the nuclei of adjacent normal mucosal epithelia, whereas no or weak staining were detected in GC tissues (Fig. 2A). 79 (87.8%) of 90 normal mucosa and 57 (63%) of 90 GC specimens exhibited high SMG‐1 expression levels (Table 1, *P* = 0.0002),and IHC signals in GC tissues were significantly lower than in normal tissues (Fig. 2A; paired *t*‐test, *P* = 0.0004). By Kaplan–Meier curve assessment, it was observed that SMG‐1 protein level was not a significant prognostic factor for poor overall survival in GC patients (Fig. 2B). However, analyses of clinicopathologic characteristics showed that expression of SMG‐1 was negatively associated with tumor size and serosal invasion (*P* < 0.05). Finally, no significant associations were found between SMG‐1 expression and age, gender, differentiation, lymphatic metastasis, tumor stage, or remote metastasis (Table 1).

**Table 1:** Clinicopathologic characteristics of GC patients

Features	*n*	High expression	Low expression	*P*
Histology				
Normal	90	79	11	***0.0002***
GC	90	57	33	
Age				0.3710
<55	25	14	11	
>=55	67	43	22	
Gender				2.3300
Male	53	37	16	
Female	37	20	17	
Tumor size				***0.0440***
<5 cm	34	22	12	
>=5 cm	56	35	21	
Differentiation				1.4420
Well	1	1	0	
Moderate	20	15	6	
Poor	69	41	27	
Serosal invasion				***0.0030***
N	8	5	3	
Y	82	52	30	
Lymphatic metastasis				0.6170
N	23	13	10	
Y	67	44	23	
Remote metastasis				2.4240
N	86	53	33	
Y	4	4	0	

The bold italics means significance of the p value.

### SMG‐1 modulates the cell proliferation and invasion induced by oncogenic miR‐192 and ‐215

**Figure 3:**
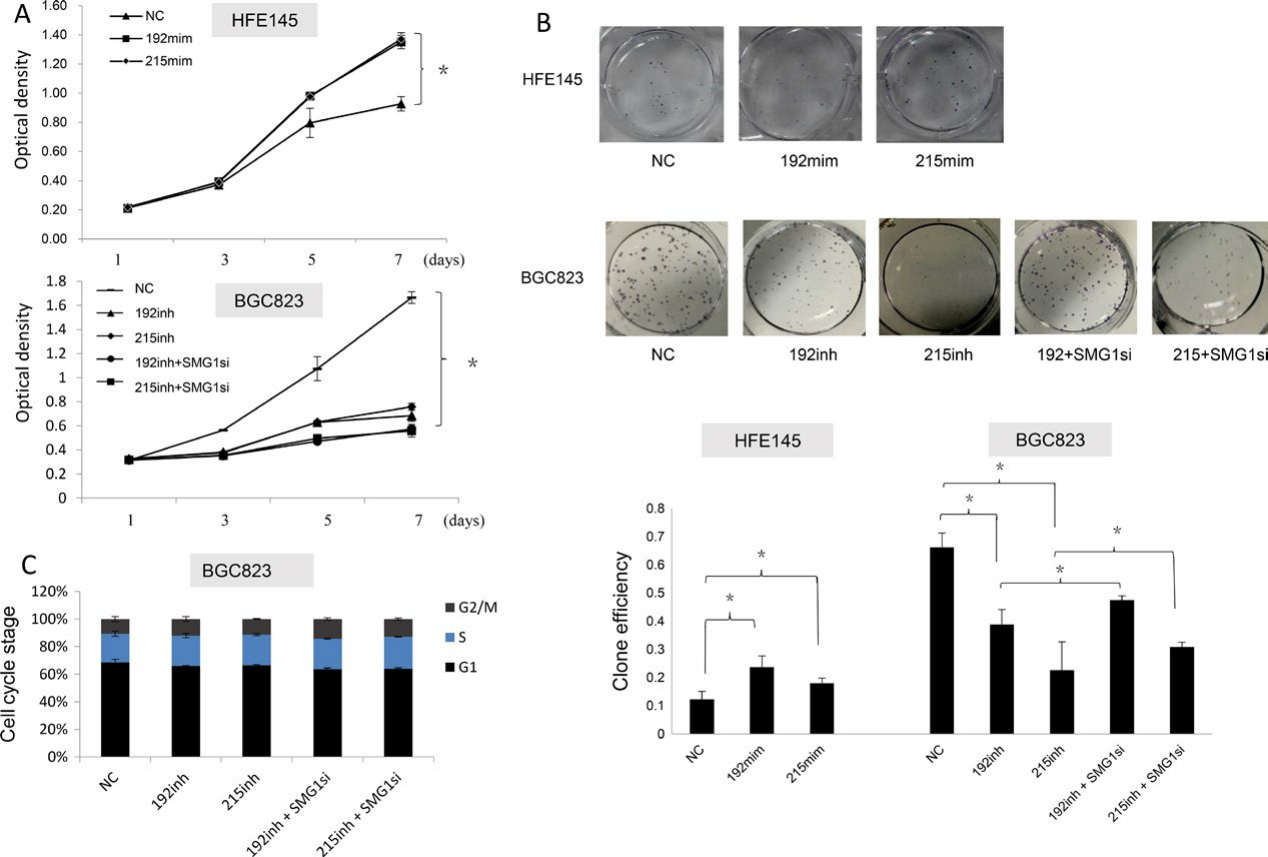
Effects of SMG‐1 regulation by miR‐192 and ‐215 on proliferation and cell cycle progression in GC cells. (A) MTT assays showed that miR‐192 and ‐215 increased proliferation. (B) Colony formation assays. MiR‐192 or ‐215 mimics increased colony formation ability in HFE145 cells, whereas decreased expression of miR ‐192 or ‐215 inhibited this ability in BGC823 cells. However, lower expression of SMG‐1 induced by a specific siRNA reversed effects on colony formation of BGC823 cells by miR‐192 and ‐215 inhibitors. (C) Cell cycle testing indicated no changes on cell cycle progression when cells were treated with miR‐192 or ‐215 inhibitors and a specific SMG‐1 siRNA. Assays were repeated three times, with triplicates performed in each independent experiment. NC, nonsense control; mim, mimic; inh, inhibitor; SMG1si, SMG‐1siRNA.

To explore the effects on proliferative properties of SMG‐1 mediated by miR‐192 and ‐215, we performed MTT and colony formation assays. Ectopic expression of miR ‐192 and ‐215 promoted cell proliferation in HFE145 cells, as shown by MTT and colony formation assays (*P* < 0.05; Fig. 3A and B). Conversely, BGC823 cells transfected with inhibitors of miR‐192 or ‐215 exhibited a significant decrease in growth rates (*P* < 0.05; Fig. 3A and B). Coincidently, inhibition of SMG‐1 by an siRNA promoted proliferation of BGC823 cells, an effect which was in turn counteracted by miR‐192 and ‐215 inhibitors (*P* < 0.05; Fig. 3A and B), suggesting that miR‐192 and ‐215 enhance growth by downregulating SMG‐1 in gastric epithelial cells. To better understand whether miR‐192 and ‐215 influence the cell cycle of GC cells via SMG‐1, we conducted cell cycle assays, using flow cytometry. Results showed that there was no effect on cell cycle progression when BGC823 cells were transfected with these miR inhibitors (Fig. 3C).

**Figure 4:**
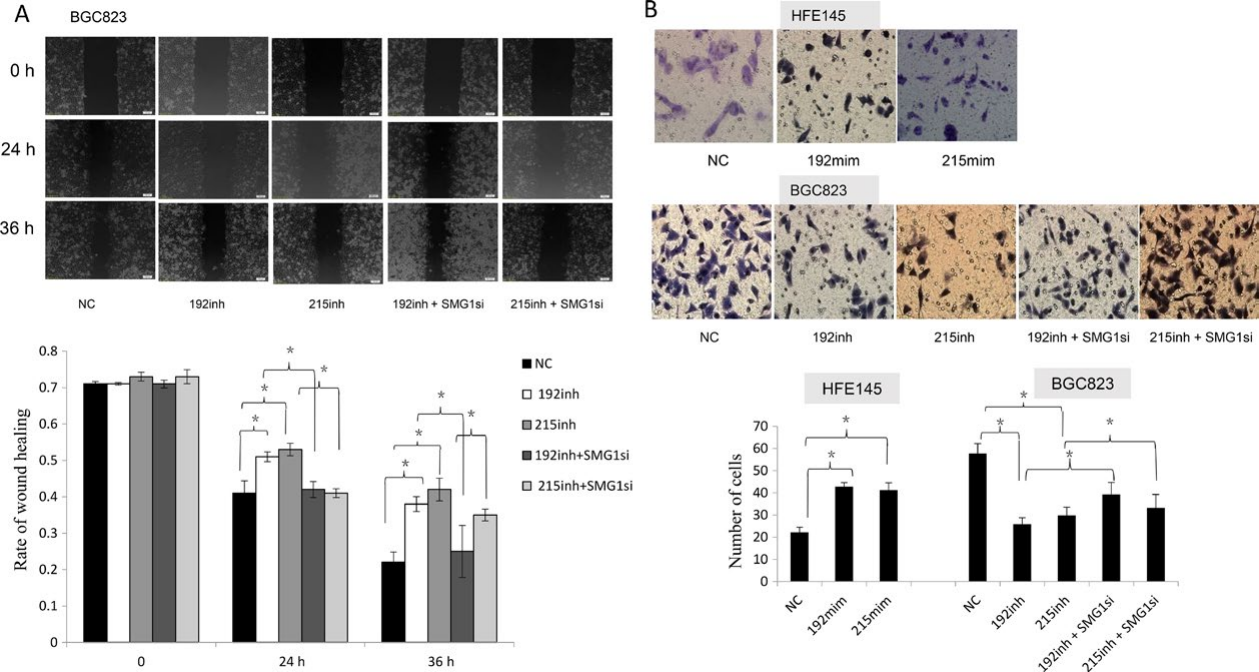
Effects of SMG‐1 regulation by miR‐192 and ‐215 on motility in GC cells. (A) Effect of miR‐192 and ‐215, which target SMG‐1, on cell migration by scratch assay. (B) Effect of miR‐192 and ‐215 on cell invasion by Boyden chamber assay. Morphologic comparison of cells penetrating the artificial basement membrane is also shown. Scale bars represent 50 *μ*m. Data represent mean ± SD. NC, nonsense control; mim, mimic; inh, inhibitor; SMG1si, SMG‐1 siRNA.

We next investigated the effects of miR‐192 and ‐215 and SMG‐1 siRNA on cell motility and invasiion. As shown in Figure 4, overexpression of miR‐192 or ‐215 enhanced motility and invasion of HFE145 cells by scratch assays and Transwell assays, respectively. Conversely, inhibition of miR‐192 or ‐215 suppressed cell migration (*P* < 0.05, Fig. 4) and invasiveness (*P* < 0.01, Fig. 4) compared with controls in BGC823 cells. Furthermore, a specific SMG‐1 siRNA promoted motility and invasiion of BGC823 cells transfected with miR‐192 or ‐215 inhibitors.

**Figure 5:**
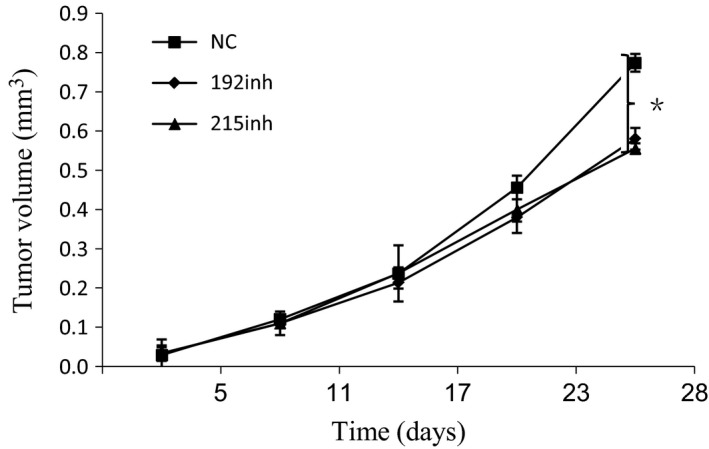
MiR‐192 and ‐215 inhibitors decrease proliferation of GC cells *in vivo*. Mice were injected with BGC823/miR‐192 and ‐215 inhibitor‐containing cells. Subcutaneous tumor volume was analyzed by Repeated Measures. MiR‐192 and ‐215 inhibitors significantly decreased tumor volumes (*P* < 0.05). NC, nonsense control; mim, mimic; inh, inhibitor.

To assess the effects of miR‐192 and ‐215 on tumor growth in vivo, BGC823 cells transfected with miR‐192 and ‐215 inhibitors or control cells were implanted subcutaneously into nude mice. Tumor size was measured by vernier caliper in primary tumors. Among these groups, mice injected with BGC823/miR‐192 and ‐215 inhibitor‐containing cells developed smaller tumors than did the NC group (*P* < 0.01) (Fig. 5).

### Activation of Wnt signaling is induced by miR‐192 and ‐215 via inhibition of SMG‐1, leading to EMT

**Figure 6:**
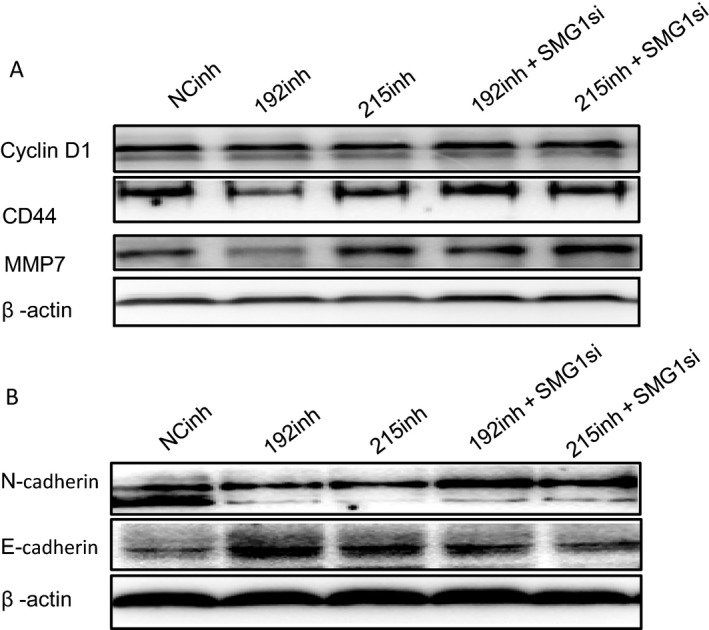
MiR‐192 and ‐215, by inhibiting SMG‐1, stimulate Wnt signaling and induce EMT in BGC823 cells. (A) Wnt signaling pathway protein expression. Inhibitors of miR‐192 and ‐215 decreased protein levels of cyclin D1, CD44 and MMP‐7. In contrast, inhibition of SMG‐1 by a specific siRNA dramatically enhanced expression of cyclin D1, CD44 and MMP‐7 proteins. (B) EMT protein expression. N‐cadherin and E‐cadherin were measured by Western blotting. Expression of N‐cadherin decreased in BGC823 cells transfected with miR‐192 and ‐215 inhibitors. Co‐transfection miR‐192 and ‐215with an SMG‐1 siRNA reversed this decreased expression. Inhibitors of miR‐192 and ‐215 increased expression levels of E‐cadherin in BGC cells. However, this enhanced expression of E‐cadherin was suppressed when an SMG‐1 siRNA was co‐transfected. NC, nonsense control; mim, mimic; inh, inhibitor; SMG1si, SMG‐1 siRNA.

The oncogenic signaling pathways, such as the Wnt signaling pathway, are associated with tumor cell proliferation, invasion, and migration 9. To elucidate signaling mediated by SMG‐1 and its regulation by miR‐192 and ‐215, we measured protein levels of the Wnt downstream targets cyclin D1, CD44, and MMP‐7 when SMG‐1 was increased by transfection of miR‐192 and ‐215 inhibitors and co‐transfected with SMG‐1 siRNA in BGC823 cells. Inhibitors of miR‐192 and ‐215 markedly decreased levels of cyclin D1, CD44, and MMP‐7 (Fig. 6A). In contrast, cyclin D1, CD44, and MMP‐7 protein levels were dramatically upregulated after co‐transfection with SMG‐1 siRNA, compared with miR‐192 and ‐215 inhibitors (Fig. 6A). It is known that Wnt signaling directly leads to heterogeneous expression of epithelial–mesenchymal transition (EMT)‐associated factors, such as N‐cadherin and E‐cadherin 10. Epithelial cells express high levels of E‐cadherin, whereas mesenchymal cells express high levels of N‐cadherin and lose E‐cadherin expression 10. Therefore, N‐cadherin and E‐cadherin proteins were measured. Compared with the NC group, N‐cadherin expression markedly decreased in BGC823 cells transfected with miR‐192 and ‐215 inhibitors (Fig. 6B); co‐transfection of miR‐192 and ‐215 inhiitors with a specific SMG‐1 siRNA reversed these effects, and N‐cadherin expression increased (Fig. 6B). E‐cadherin exhibited increased expression in BGC823 transfected with miR‐192 and ‐215 inhibitors (Fig. 6B). The enhanced expression of E‐cadherin in BGC823 cells transfected with miR‐192 and ‐215 inhibitors was suppressed when an SMG‐1 siRNA was co‐transfected. Taken together, these findings indicate that miR‐192 and ‐215 promote the development of GC, at least inpart, via activating Wnt signaling and EMT by targeting SMG‐1.

## Discussion

GC is one of the most common malignancies in the Asia‐Pacific region, especially in China 11, 12. For most patients, there are no specific clinical symptoms during its early stages. Although much preclinical and clinical research has focused on the importance of GC, clinical application of these findings to achieve improved outcomes has not been effectively achieved. Therefore, the identification of novel biomarkers and underlying mechanisms regulating gastric cancer progression are key steps in future diagnostic and therapeutic strategies.

SMG‐1 is known as a critical component of an evolutionarily conserved mRNA surveillance mechanism, NMD 13. It is also well known that SMG‐1 participates in the regulation of normal cell growth, TNF*α*‐induced apoptosis 2, and cell cycle progression, all of which are important mechanisms of carcinogenesis 13. SMG‐1 was discovered to be involved in the development of several different tumor types 2, 3, 14. Thus, SMG‐1 has been suggested as a tumor suppressor gene, although its role as an NMD effector has been well documented. However, the involvement of SMG‐1 in the development of GC has not yet been explored. In this study, we analyzed the expression of SMG‐1 in GC tissues. We found that SMG‐1 protein levels were significantly lower in GC specimens than in normal tissues. This finding suggests that SMG‐1 is a candidate tumor suppressor gene in GC. Similar observations have been made by other groups in other cancer types. For example, Gubanova et al. 2 showed that SMG‐1 suppressed oncogenic CDK2‐driven proliferation and functioned as a tumor suppressor in osteosarcoma and colorectal tumors. Similarly, Evgenia 3 found that SMG‐1 in HPV‐positive head and neck squamous cell carcinoma was downregulated due to promoter hypermethylation. In hepatocellular carcinoma, SMG‐1 was also underexpressed and suggested as a biomarker for early‐stage patients. Finally, another recent study demonstrated that SMG‐1 regulates the p53 and Cdc25A signaling pathways to suppress tumor growth and functions as a tumor suppressor gene in acute myeloid leukemia, osteosarcoma and cancer 2, 8, 14, 15. Taken together, these findings support SMG‐1 as a tumor suppressor gene in cancer.

MiR‐192 and ‐215 are closely related, with similar seed sequences, and have been increasingly suggested as onco‐ or tumor suppressor miRs in many tumor types, including renal childhood neoplasms 7, colorectal cancer 8, osteosarcoma 15, and esophageal squamous cell carcinoma 16. Yousef 7 found that upregulation of miR‐192 and ‐215 decreased kidney cancer cell proliferation, migration, and invasion. In colorectal cancer (CRC) tissues, miR ‐192 and ‐215 were found to be significantly downregulated and were associated with increased tumor size 8. In a previous study, we showed that miR‐192 and ‐215 were oncogenic miRs in GC and functioned by targeting ALCAM 5.

In this study, based on previous knowledge of miR ‐192 and ‐215, we hypothesized that targets of miR ‐192 and ‐215 could be explored as novel biomarkers of GC. Therefore, we sought to predict and identify targets of miR‐192 and ‐215 in GC by using gene microarray assays and in silico prediction engines, which identified SMG‐1 as a strong candidate target. Luciferase activity assays confirmed that miR‐192 and ‐215 directly bound to the 3′‐UTR of SMG‐1. Moreover, low expression levels of SMG‐1 in GC samples supported a tumor‐suppressive role in GC and correlated with clinical parameters. Furthermore, as far as biological functions are concerned, miR‐192 and ‐215 increased proliferation, migration and invasion in GC cells, while SMG‐1 siRNA reversed the effects of miR‐192 and ‐215. Animal model assays showed that miR‐192 and ‐215 inhibitors decreased proliferation of GC cells in vivo*,* as well. Taken together, these findings suggested that SMG‐1 modulation by miR‐192 and ‐215 is a functional tumorigenic pathway in GC, in agreement with findings for other tumor types 2, 3, 14, 17.

Currently, emerging studies suggest that activation of the Wnt pathway contributes to carcinogenesis in at least a subset of GCs 18, 19, 20, and that Wnt signaling plays a key role in the progression of EMT 20. Moreover, abnormal EMT activation is associated with GC 21. Therefore, studies of regulatory mechanisms underlying GC have been increasingly focused on dysregulated Wnt pathway protein levels 20. Guo 22 et al. found that PRRX1 was upregulated and promoted EMT through the activation of Wnt signaling in GC cells. Additionally, EphA2 was found to promote EMT via the Wnt/beta‐catenin pathway in GC cells 20. EMT led to carcinogenesis and metastasis and was recognized as an important step in invasion and metastasis 23. Until now, there has been only one report showing that ectopic expression of miR‐192 and ‐215 increased E‐cadherin levels via repressed translation of ZEB2 mRNA in renal cells 24. In addition, it is known that miRs can activate or inhibit canonical Wnt signaling at multiple levels by targeting Wnt‐associated proteins 25. Based on these previous results, we hypothesized that SMG‐1 regulation by miR‐192 and ‐215 targeted EMT that was in turn mediated by the Wnt signal pathway. In order to ascertain whether SMG‐1 targeted the Wnt pathway activity to mediate EMT, Wnt pathway‐associated proteins were examined. These assays proved that inhibitors of miR‐192 and ‐215 markedly decreased expression of the Wnt pathway proteins Cyclin D1, CD44, and MMP‐7. Notably, a specific SMG‐1 siRNA reversed these effects. Thus, we concluded that SMG‐1 appeared to mediate gastric carcinogenesis through the Wnt pathway. Meanwhile, we also examined expression of the mesenchymal protein N‐cadherin and the epithelial‐specific junction protein E‐cadherin. Results illustrated that loss of function of miR‐192 and ‐215 caused decreased expression of N‐ cadherin, while miR‐192 and ‐215/SMG‐1 siRNA reversed this inhibitory effect. In contrast, E‐cadherin exhibited increased expression levels in the miR‐192 and ‐215 inhibitor group. Therefore, we concluded that SMG‐1 inhibition by miR‐192 and ‐215 promotes the GC development by stimulating EMT.

In summary, we have now shown that SMG‐1 is a target of the oncomiRs, miR‐192 and ‐215, and that its downregulation is associated with tumor size and serosal invasion of GC. In addition, we have demonstrated that miR‐192 and ‐215 promote cell proliferation and migration in GC, at least in part, by activating Wnt signaling via targeting of SMG‐1. Moreover, SMG‐1 appears to mediate at least some of the oncogenic function of miR‐192 and ‐215 by participating in EMT, triggered by activation of the Wnt pathway in GC. Our characterization of this signaling pathway contributes to a deeper understanding of the development and progression of GC, and may provide future therapeutic targets for the treatment of GC.

## Conflict of Interest

The authors disclose no potential conflicts of interest.

## Supporting information


**Table S1.** SMG1 primer sequences.
**Table S2.** Effects of miRs‐192/‐215 target genes by microarray and alignment prediction.
