# Supplementary material for: SMG‐1 inhibition by miR‐192/‐215 causes epithelial‐mesenchymal transition in gastric carcinogenesis via activation of Wnt signaling
Source: Cancer Med. 2017 Dec 13;7(1):146–56. doi: 10.1002/cam4.1237 (PMC5773975; doi:10.1002/cam4.1237)
Supplement: Supplementary file 1 — Table S1. SMG1 primer sequences. Table S2. Effects of miRs‐192/‐215 target genes by microarray and alignment prediction. [file CAM4-7-146-s001.pdf]

Supplementary Table 1. SMG1 Primer Sequences

**Sequences of siRNA**	
SMG1-siRNA-F	5’GUGUAUGUGCGCCAAAGUAdTdT 3’
SMG1-siRNA-R	3’dTdT CACAUACACGCGGUUUCAU 5‘
**Construction of Plasmids**	
SMG1-3UTR-F	CACCTCGAGATGGCAAGACAGTAGATGAGTCTGG
SMG1-3UTR-R	AAATATGCGGCCGC TCTCGCTCTCCTAACCTCATGATCC
SMG1-mut-F	CAGTAGATGAGTCTGGTTAAGCGTCCAGTCACATCCACCAGAATCAACTCAGC
SMG1-mut-R	GCTTATAGTGTGCATTTTTTTTTGGTCCAGTTTCAAATTTTCCCTCTTCTGTG

Supplementary Table 2. Effects of miRs-192/-215 target genes by microarray and alignment prediction

	Target Gene Expression on Microarray			Target Gene Prediction		
Cell line	GeneSymbol	Fold change ([192&215] vs [NC] >= 2.0)	Regulation	miRanda	PICTAR4	SUM
BGC823/192i	SMG1	6.500	up	1	1	2
BGC823/215i	SMG1	4.523	up			
HFE145/192m	SMG1	3.033	down	1	1	2
HFE145/215m	SMG1	4.152	down			

hsa-miR-192/SMG1 Alignment

```
   3' ccgacaguuaaguaUCCAGUc 5' hsa-miR-192
                    ||||||
19:5' agucugguuaagcgAGGUCAg 3' SMG1
```

mirSVR score: -0.0028
PhastCons score: 0.8318

hsa-miR-215/SMG1 Alignment

```
   3' cagacaguuaaguaUCCAGUa 5' hsa-miR-215
                    ||||||
19:5' agucugguuaagcgAGGUCAg 3' SMG1
```

mirSVR score: -0.0028
PhastCons score: 0.8318

MiRNA target-predicting algorithms were used to predict targets of miR-192/215 based on the presence of binding sites in their 3'UTRs. Abbreviations: GC, gastric cancer; 192i, miR-192 inhibitor ; 192m, miR-192 mimic; 215i, miR-215 inhibitor; 215m, miR-215 mimic; NC, nonsense control
